# The Role of Age and Multimorbidity in Shaping Older African American Men’s Experiences with Patient–Provider Communication

**DOI:** 10.3390/geriatrics3040074

**Published:** 2018-10-24

**Authors:** Ramona G. Perry, Jamie A. Mitchell, Jaclynn Hawkins, Vicki Johnson-Lawrence

**Affiliations:** 1 School of Social Work, University of Michigan School of Social Work, Ann Arbor, MI 48103, USA; ramonagp@umich.edu (R.G.P.); jachawk@umich.edu (J.H.); 2 College of Human Medicine, Michigan State University, Flint, MI 48502, USA; vicki.johnson-lawrence@hc.msu.edu

**Keywords:** communication, comorbidity, men, African American

## Abstract

This study investigated factors associated with older African American men’s unmet health communication needs in the context of patient–provider interactions. Responses to a health survey were analyzed for 430 African American men attending a Midwest community health fair. The outcome measure was the extent to which men could get their health-related questions answered during recent medical visits. Men’s mean age was 54; 39% had one chronic condition and 22% had two or more comorbidities. The 53% who usually or always had their questions answered were older, had less comorbidity, higher educational attainment, higher annual incomes, were more likely to be married and have any type of insurance, and have a personal physician. Access to care was the primary factor in shaping men’s opportunities to ask health-related questions, and older multimorbid and low-income African American men may face increased barriers to healthcare access, and thus barriers to patient-centered care and communication.

## 1. Introduction

African American men persistently experience higher levels of multimorbidity (i.e., two or more chronic medical conditions) as they age, in comparison to White and Hispanic men [1]. Managing multiple chronic conditions often necessitates more frequent communication with health care providers, and research has shown that many African American men report suboptimal experiences with medical professionals, which may negatively affect their engagement in positive health behaviors and medical decision making [2,3]. For example, one study by Griffith, Ober Allen & Gunter [3] documented that some African American men felt particularly uncomfortable with the tone physicians used in communicating with them during medical visits and reported that while physicians made recommendations for their care, they rarely discussed specific strategies for meeting or implementing those recommendations. Another study reported that compared to White lung cancer patients, African American patients tended to judge communication with their physicians’ as less informative, less supportive, and less collaborative, resulting in lower trust in the physician [4]. Older African American men in particular may be missing opportunities to benefit from the type of patient-centered and culturally informed patient–provider communication that is associated with increased patient satisfaction, improved medication adherence, and greater uptake of provider-recommended management behaviors, specifically among those with chronic diseases [5].

One strategy to improve health outcomes among populations with disproportionate chronic disease risk has been to encourage question asking during medical visits [6]. Patient-centered communication can be defined as communication that prioritizes the patient’s perspective, preferences, and psychosocial wellbeing. Literature suggests that physicians engage in significantly more patient-centered communication when interacting with patients who participate to a high degree, compared with patients who are less engaged [6]. However, the extent to which older African American men actively participate (i.e., question asking) during medical visits is unclear. Even less is known about whether older African American men with multimorbidity are consistently experiencing high-quality patient–provider communication. This is particularly important because the burden of multimorbidity is higher among older adults, men, and racial/ethnic minority groups [7]. Much of the existing literature connecting multimorbidity to health communication among African Americans centers on Type 2 diabetes, a condition that increases the risk for other chronic illnesses such as hypertension, kidney disease, and cardiovascular disease, and is prevalent among African Americans [8]. Among older African American men specifically, the health communication knowledge base is somewhat limited to studies on prostate cancer, a disease where African American men are diagnosed with more advanced disease and have poorer long-term survival than White men [9]. Age also plays a critical role in shaping the health communication dynamics for older adults, especially those with multimorbidity. For instance, one observational study demonstrated that some older adults experienced shorter primary care medical visits and less patient-centered communication, even when a companion was present to assist them [10]. This intersection of race, gender, age, and multimorbidity is underexplored in extant literature. Specifically, few studies have been identified that capture the intersection of health communication difficulties for older African American men with multimorbidity. The current study addresses this gap by investigating the extent to which age and multimorbidity are associated with African American men’s unmet health communication needs in the context of patient–provider interactions. This work is informed by an empowerment model of health promotion focused on supporting the preparation and capability of patients to make informed choices about their health, a complimentary concept and practice to patient-centered care and communication [11].

## 2. Methods

This study was a secondary analysis of a data set originally collected to capture African American men’s general health-related beliefs and behaviors. The institutional review board of the Cleveland Clinic Foundation approved the original study in March 2011 under protocol #11-199. Participants were a purposive sample of African American men aged 18 and older who attended a community health fair geared toward minority men and hosted by the Minority Men’s Health Center within the Cleveland Clinic Foundation. Participants, who represented 21.5% of the approximately 2000 health fair attendees, included 430 men with non-missing data on all measures of interest. For the original study, a team of trained graduate research assistants approached potential participants waiting in line to register for the event and after listening to an approved script and providing verbal consent, participants completed the survey on clipboards while in line. Research assistants were also stationed throughout the event to collect completed surveys. Participants in the original study completed the 40-item survey to characterize their health behaviors and habits, health conditions, physician interactions, and other health related information. The outcome measure for this secondary analysis was the extent to which patients could get their health-related questions answered. This outcome was assessed by the item “How often did doctors, nurses, or other health professionals give you the chance to ask all the health-related questions you had [in the past 12 months].” Responses to this item included “never”, “sometimes”, “usually”, and “always.” For the purposes of analysis this measure was recoded into a binary indicator where “never” and “sometimes” were coded as “no” and “usually” and “always” were recoded as “yes”. This item was adapted from an extensive theoretical body of work on patient participation in medical visits, including the centrality of patient question asking to patient satisfaction, adherence to treatment recommendations, trust, and engagement in medical decision-making [6].

The primary independent variable was the number of comorbid chronic conditions. Respondents were asked if they had ever been told by a doctor or health care professional that they had any of six chronic health conditions: Any heart condition, hypertension, diabetes, kidney disease, high cholesterol, and cancer. The measure for number of comorbidities used in analyses was created by combining these six conditions to create a measure of count, ranging from 0–5 chronic conditions. Owing to the relatively low frequency of participants with 3–5 comorbidities, these categories were collapsed into “3 or more” comorbidities. Several demographic characteristics of patients were also identified for inclusion as covariates in nested binary logistic regression analyses. Age was measured continuously and ranged from 18 to 90 years ($\bar{x}=54.35$, SD 12.9); 67.4% of the sample was over age 50 and less than 7% was age 35 or younger (see Table 1). Education level was measured through an item with the following options: less than elementary, less than high school, high school diploma or GED, some college, college graduate, and advanced degree. For analytic purposes, less than high school and less than elementary school were combined. Other demographic covariates included annual income and marital status. Measures indicative of healthcare access were also included in analyses. Insurance coverage was measured through a binary (1) yes or (0) no response to having any insurance. Respondents were asked if they had someone that they considered to be their personal physician and they could answer (1) yes, (2) more than one, or (3) no doctor. We also included the location respondents sought medical care most often from (e.g., (1) doctor’s office, (2) emergency room, (3) outpatient/urgent care, or (4) some other place).

Secondary analysis was performed on an analytic sample of 430 African American men using STATA/SE 15 (2017). Patients’ clinical (number of comorbidities) and demographic (education, age, income, marital status) characteristics, as well as health care access factors (i.e., insurance status, place of care, personal physician) were described using descriptive statistical techniques (e.g., frequencies, mean, SD). Bivariate differences between respondents who reported getting their health-related questions answered and those who reported not getting their questions answered were tested with Chi-square and ANOVA. A nested binary logistic regression was conducted to determine multimorbidity, age, and demographic factors, and health care access factors were predictors of getting health related questions answered. Model 1 included age and number of chronic illnesses (0, 1, 2 or 3 or more). Model 2 added education (less than high school, high school graduate, some college, college graduate, advanced), income, and marital status. Model 3 added insurance status (yes or no to having insurance), whether respondent had a primary physician (one, more than one, or none), and place respondent usually sought care (doctors office, ER, outpatient, or some other place).

## 3. Results

The analytic sample (*n* = 430) included participants for whom there were no missing values on measures of interest. A little over half of respondents (53%) reported usually or always getting their questions answered, whilst 47% reported never or sometimes. Over three-fourths of respondents reported having one or fewer chronic illnesses, with the remaining respondents reporting 2 or more comorbidities. The sample characteristics are summarized in Table 1.

The present study found statistically significant bivariate differences between patients who had their questions answered and those who did not. Patients who were able to ask their health-related questions were older (56 compared to 51 years, F (1428) = 15.67, *p* < 0.001) and had less comorbidity on average (x^2^ = 16.93, *p* < 0.01). They also had higher educational attainment, higher annual incomes, and were more likely to be married (see Table 2). Healthcare access factors were also significantly associated with getting questions answered. Nearly 70% of respondents who reported having any type of insurance had their health-related questions answered, compared to 30% of those who reported lacking insurance (x^2^ = 61.03, *p* < 0.001). Having a personal physician was associated with getting questions answered with 70% of those with one or more than one physician, compared to 32% of those with no physician. Finally, nearly three fourths of those who received care in a doctors’ office reported being able to get their health-related questions answered, compared to under a third of those who received care in a hospital ER.

In light of the bivariate differences between respondents who were and were not able to get their health related questions answered, we conducted a series of nested binary logistic regression models predictive of respondent’s report of being able to get their health related questions answered (see Table 3). Models 1, 2, and 3 accounted for 8.3%, 16.2%, and 31.4% of the variability in getting health related questions answered, respectively. In the first nested binary logistic regression model, age and number of chronic illnesses were significant predictors; those who had three or more comorbidities had three times higher odds of getting their questions answered, relative to those who had no chronic illnesses (OR = 3.02, 95% CI:1.26, 7.23, *p* < 0.05). Moreover, as age increased, so did the odds of having questions answered (OR = 1.02, 95% CI:1.01, 1.04, *p* < 0.01). In model 2, education, income, and marital status were added as control measures. Age and number of comorbidities remained significant with comparable odds ratios to model 1 (OR = 1.02 and 2.95, respectively). Education and marital status were not predictive of getting questions answered whilst income was, such that those with income levels less than $25,000 had a third of the odds of getting their questions answered compared to those who made over 50 k annually.

However, when controlling for insurance status, personal physician, and location of health care services in Model 3, age, number of comorbidities, and income lost significance. Those who reported having no insurance coverage had 56% lower odds of getting their questions answered, compared to those who reported having any kind of insurance (OR = 0.44, 95% CI: 0.24, 0.78, *p* < 0.01). Similarly, those who reported not having a personal physician had 56% lower odds of getting their questions answered as those who had a personal physician (OR = 0.44, 95% CI: 0.24, 0.79, *p* < 0.01). Finally, location of health care services was also a significant predictor; the odds of getting questions answered were 60% lower among those seeking healthcare in a hospital ER compared with those who sought care in a doctor’s office.

## 4. Discussion and Conclusions

This study explored the roles of multimorbidity and age in relation to getting health questions answered among African American men over age 50. With 62% of the U.S. population of older adults currently managing two or more chronic conditions [12,13] and 48% having three or more conditions [1], the health and quality of life of aging adults will depend ever more substantially on high quality doctor-patient communication. Furthermore, there is a current drive to improve clinical management of chronic conditions in an effort to reduce health care costs. Increased opportunities for patients to raise more frequent and detailed concerns in fewer clinical encounters may be one strategy to achieve that goal. First, participants that reported having no insurance coverage, those who reported not having a personal physician, and those who more routinely utilized hospital emergency departments all had lower odds of getting their questions answered by a health provider. These findings underscored how the fragmented healthcare experiences of these men are deeply connected to the primacy of health care access. For example, health insurance coverage is generally a strong positive correlate with access to a personal primary care physician [14]. Multiple dimensions of healthcare access emerged as foundational to addressing unmet health information needs for the aging African American men in this study. African American men who lack access to insurance as a gateway into the healthcare system are set up to experience a domino effect of challenges that stultifies their agency as health care consumers, including the ability to ask questions and actively engage in managing their own care.

Second, while number of comorbidities did not bear out as a significant factor in the final model, it is noteworthy to consider the significant bivariate findings for participants in this study. Specifically, significantly more participants with only one chronic illnesses reported getting questions answered compared to participants with two or three comorbidities. There are several potential underlying reasons why someone with more chronic health needs may have less of an opportunity to pose questions related to their care. For instance, well-noted time constraints during medical visits where several conditions must be examined and managed could leave less time to address patient questions than visits where fewer health needs are being attended. Relatedly, medical visits for multiple chronic conditions may require the provider to consult or otherwise interact with nurses, pharmacy or lab personnel, and even health insurance or billing personnel regarding medical tests, medication-related concerns, and follow-up care more so than during visits that are less medically complex. These care-related realities may have the unintended consequence of reducing much-needed opportunities for African American male patients to engage with the healthcare provider and foreground their specific concerns during the encounter.

Again, while not significant in the final model, it is important to consider how African American male participants who were able to ask their health-related questions were older in bivariate analyses. From a lifespan perspective, African American men over age 50 may get more questions answered during clinical encounters because they have greater confidence and experience in health information seeking behaviors than their younger counterparts [13]. In addition, aging adults may be more likely to see providers that address their collective biopsychosocial health needs (e.g., geriatricians); access to aging specialists may increase the patient’s ability and provider’s competence to approach complex questions related to overall disease management, particularly for those with limited functional abilities. While the current study was not able to tease apart the influence of companions or family members in influencing question asking during medical visits, it is interesting to note that aging adults with chronic diseases are more likely to have family members involved in managing their care than younger adults, and that support network likely plays some role in facilitating more detailed information exchange during clinical visits [1]. Among non-elderly African American men, multimorbidity is often more severe, with increased complications and risk for mortality [1,15]. This trend should signal the need for improved access to care overall, and better disease management specifically, including higher quality patient–provider communication for non-elderly men. Unfortunately, there has not been a significant health care focus on resources for and attention to multimorbidity for this population.

We interpret our findings with caution given certain limitations. Specifically, the sample size may limit the power of our study to detect associations between multimorbidity and question asking particularly among the non-elderly samples. While theoretically derived, the outcome measure has not been rigorously tested for validity. The cross-sectional survey was administered at a single point in time and does not reflect the specific health conditions participants may have asked about with their providers and does not allow us to make causal inferences about the associations found in our analyses. In addition, the sample of respondents in these analyses was drawn from a localized hospital-based health fair; men who may be more health-seeking than the broader population, which may limit the generalizability of our study findings to other samples of African American men. Without knowledge of the frequency or recency of primary care visits in the prior twelve months, it is difficult to infer whether some men may have had difficulty recalling the experience of question asking for visits that were closer to 12 months prior.

These limitations notwithstanding, these findings suggest that the ability of aging African American men to engage in patient-centered communication is significantly shaped by access to care primarily, and that age and degree of multimorbidity may be other important indicators in need of further attention. Results highlight a need for health care professionals, particularly in primary care settings, to develop a mechanism to both assess and improve the quality of patient–provider communication, considering how aging patients with complex health needs could be better served. Research suggests that a team approach to health care delivery is one way to improve health outcomes for older adults, whilst also improving communication between health care providers treating the same patient for different conditions [16,17]. This is particularly important for aging African American men with multiple chronic illnesses. As our study suggests, health care professionals should also keep in mind that African American men who are uninsured, do not have a personal physician and those who seek regular healthcare in hospital emergency rooms may be at an increased risk of not getting their questions answered by physicians. Additional research is needed to clarify any potential interactions between older age and the increased prevalence of specific chronic conditions in relation to unmet communication needs for this population. Further, there is a need for new and innovative strategies that both support clinicians in better communicating with aging African American patients, and that empower patients to advocate for the highest quality patient-centered care.

## Figures and Tables

**Table 1:** Univariate Frequencies and descriptive of measures of interest, MMHS, 2011. Analytic Sample: African American Males.

	N *Total* *n* = 430	% 100%
**Primary Dependent Variable**		
chance to ask questions ^a^		
Yes	229	53.26
No	201	46.74
**Primary Independent Variable**		
number of comorbidities ^b^		
0	165	38.37
1	170	39.53
2	60	13.95
3 or more	35	8.14
covariates		
**Demographic Factors**		
age years (mean)		
	54.35	12.39 (SD)
age categories		
under 35 years	29	6.74
36–49 years	111	25.81
50–64 years	206	47.91
over 65 years	84	19.53
education		
less than high school	33	7.67
high school	171	39.77
some college	153	35.58
bachelors	52	12.09
advanced	21	4.88
income		
less than 20 K	161	37.44
20–25 K	53	12.33
25–35 K	72	16.74
35–50 K	67	15.58
greater than 50 K	77	17.91
marital status		
married	138	39.07
divorced/separated	128	29.77
widowed	18	4.19
never married/unmarried	116	26.98
**Access Factors**		
insurance coverage		
Yes	248	57.67
No	182	42.33
personal physician		
one	181	42.09
more than one	54	12.56
none/don’t know	195	45.35
place of care		
doctors office	183	42.56
hospital ER	120	27.91
hospital outpatient/urgent care	70	16.28
other place/don’t know	57	13.26

^a^ Yes = always/often No = sometimes/never. ^b^ Six individual illness indicators combined. 3 + = 3, 4, or 5 comorbidities.

**Table 2:** Bivariate tests of association [Chi Square] between having questions answered and covariates of interest, MHHS, 2011.

	Able to Get Questions Answered		
	No	Yes	
	Row %, (N)	Row %, (N)	x^2^, *p*
number of comorbidities **			16.93, *p* < 0.01
0	55.15 (91)	44.85 (74)	
1	40.59 (69)	59.41 (101)	
2	55.00 (33)	45.00 (27)	
3 or more	22.86 (8)	77.14 (27)	
age in years ***			18.03, *p* < 0.001
under 35 years	62.07 (18)	37.93 (11)	
36–49 years	54.05 (60)	45.95 (51)	
50–64 years	48.54 (100)	51.46 (106)	
over 65 years	27.38 (23)	72.62 (61)	
education *			12.78, *p* < 0.05
less than high school	45.45 (15)	54.55 (18)	
high school	52.63 (90)	47.37 (81)	
some college	47.71 (73)	52.29 (80)	
bachelors	38.46 (20)	61.54 (32)	
advanced	14.29 (3)	85.71 (18)	
income ***			27.83, *p* < 0.001
less than 20 K	58.93 (94)	41.61 (67)	
20–25 K	58.49 (31)	41.51 (22)	
25–35 K	43.06 (31)	56.94 (41)	
35–50 K	37.31 (25)	62.69 (42)	
greater than 50 K	25.97 (20)	74.03 (57)	
marital status *			8.08, *p* < 0.05
married	39.88 (67)	60.12 (101)	
divorced/separated	46.09 (59)	53.91 (69)	
widowed	50.00 (9)	50.00 (9)	
never married/unmarried	56.90 (66)	43.10 (50)	
insurance ***			61.03, *p* < 0.001
Yes	30.65 (76)	69.35 (172)	
No	68.68 (125)	31.32 (57)	
personal physician ***			62.89, *p* < 0.001
one	29.28 (53)	70.72 (128)	
more than one	29.63 (16)	70.37 (38)	
none/don’t know	67.69 (132)	32.31 (63)	
place of care ***			67.64, *p* < 0.001
doctors office	25.14 (46)	74.86 (137)	
hospital ER	68.33 (82)	31.67 (38)	
hospital outpatient/urgent care	48.57 (34)	51.43 (36)	
other place/don’t know	68.42 (39)	31.58 (18)	

* *p* < 0.05. ** *p* < 0.01. *** *p* < 0.001.

**Table 3:** Nested Binary Logistic Regression Predictive of Patient Ability to Get Questions Answered.

	Model 1 ***		Model 2 **		Model 3 ***	
	OR	95% CI	OR	95% CI	OR	95% CI
number of comorbidities						
0 ^†^						
1	1.55**	[0.98, 2.41]	1.44**	[0.89, 2.30]	1.11**	[0.65, 1.87]
2	0.79**	[0.42, 1.47]	0.77**	[0.40, 1.47]	0.55**	[0.26, 1.12]
3 or more	3.02 **	[1.26, 7.23]	2.95 **	[1.20, 7.29]	1.65**	[0.62, 4.31]
age in years						
	1.03 **	[1.01,1.04]	1.02 **	[1.00, 1.04]	1.00**	[0.98, 1.02]
education						
less than high school			0.44**	[0.99, 1.95]	0.55**	[0.11, 2.76]
high school			0.31**	[0.08, 1.15]	0.32**	[0.07, 1.32]
some college			0.32**	[0.08, 1.17]	0.30**	[0.07, 1.23]
bachelors			0.48**	[0.11, 1.95]	0.34**	[0.07, 1.53]
advanced ^†^						
income						
less than 20 K			0.35 **	[0.17, 0.72]	0.69**	[0.31, 1.55]
20–25 K			0.31**	[0.13,0.68]	0.59**	[0.24, 1.43]
25–35 K			0.52**	[0.24, 1.09]	0.71**	[0.31, 1.61]
35–50 K			0.76**	[0.35, 1.64]	0.96**	[0.42, 2.21]
greater than 50 K ^†^						
marital status						
married ^†^						
divorced/separated			1.08**	[0.63, 1.85]	1.39**	[0.76, 2.52]
widowed			0.61**	[0.20, 1.83]	0.61**	[0.19, 1.95]
never married/unmarried			1.00**	[0.56, 1.78]	1.37**	[0.72, 2.61]
insurance						
Yes ^†^						
No					0.44 **	[0.24, 0.78]
personal physician						
one ^†^						
more than one					1.06**	[0.51, 2.23]
none/don’t know					0.44 **	[0.24, 0.079]
place of care						
doctors office^†^						
hospital ER					0.37 **	[0.20, 0.71]
hospital outpatient/urgent care					0.62**	[0.32, 1.20]
other place/don’t know					0.41 *	[0.18, 0.91]
nagelkerke R^2^	0.083		0.162		0.314	

^†^ Reference category. * *p* < 0.05. ** *p* < 0.01. *** *p* < 0.001.
